# Unilateral Leukocoria in an Infant

**DOI:** 10.7759/cureus.11596

**Published:** 2020-11-20

**Authors:** Deepa Vasireddy, Jibran E Atwi

**Affiliations:** 1 Pediatrics, Pediatric Group of Acadiana, Lafayette, USA

**Keywords:** leukocoria, white pupillary reflex, mittendorf dot, persistent hyperplastic primary vitreous, retinoblastoma, infant

## Abstract

A nine-day-old male infant presents with his mother to the pediatric clinic with a concern of a white spot in his right eye. There was no history of antenatal or perinatal complications and the mother's serology was negative. Physical examination was remarkable only for leukocoria in the right eye. He was evaluated to have a Mittendorf dot. This is a benign clinical finding. Pupillary reflex check is an important part of a physical examination which can recognise pathological conditions such as cataract, retinoblastoma, metabolic errors with ocular manifestations and lead to early treatment before the child loses vision.

## Introduction

Infants are screened at the time of birth and periodically after for pupillary red reflex.

Leukocoria can be detected by family members or is usually picked up by the pediatrician during screening for red reflex by direct ophthalmoscopic examination. Family members tend to notice it incidentally either by direct observation of the child or in photographs taken utilizing the flash mode in which they notice a white pupil. Once detected a referral to an ophthalmologist is promptly performed for further evaluation.

There are several differential diagnoses for leukocoria. Retinoblastoma is of primary concern when leukocoria is detected. It represents about 6% of malignant tumors in children five years of age and younger [1]. It can involve one or both eyes and can lead to blindness and death due to metastasis [2]. Congenital cataracts are the leading cause of leukocoria [3]. They can occur as part of a syndrome or secondary to metabolic defects or due to intrauterine infection, drug or ionizing radiation exposure. If not treated within the first year of life, vision will not be regained post-surgical correction [4]. The incidence of retinopathy of prematurity (ROP) has been on the rise with more preterm infants getting medical care in our neonatal critical care units. ROP in stage 3 and higher stages can present with leukocoria [5]. Coats disease, the specific cause of which is unknown features abnormal development of blood vessels in the retina and can lead to blindness and may in late stages necessitate enucleation of the eye [6]. Familial exudative vitreoretinopathy is another cause of abnormal retinal vascular development [7]. Intraocular inflammation from various infections can lead to leukocoria from the debris. Retinal detachment from innumerable causes can also present with leukocoria [8].

Embryonic vascular connective tissue tends to regress by around four months of gestational age. The arrest of the regression leads to persistent hyperplastic primary vitreous (PHPV). It is one of the commonest benign lesions to cause leukocoria.

We present the case of an infant who presented with unilateral leukocoria. The patient’s identification has been kept confidential in the manuscript.

## Case presentation

**Figure 1:**
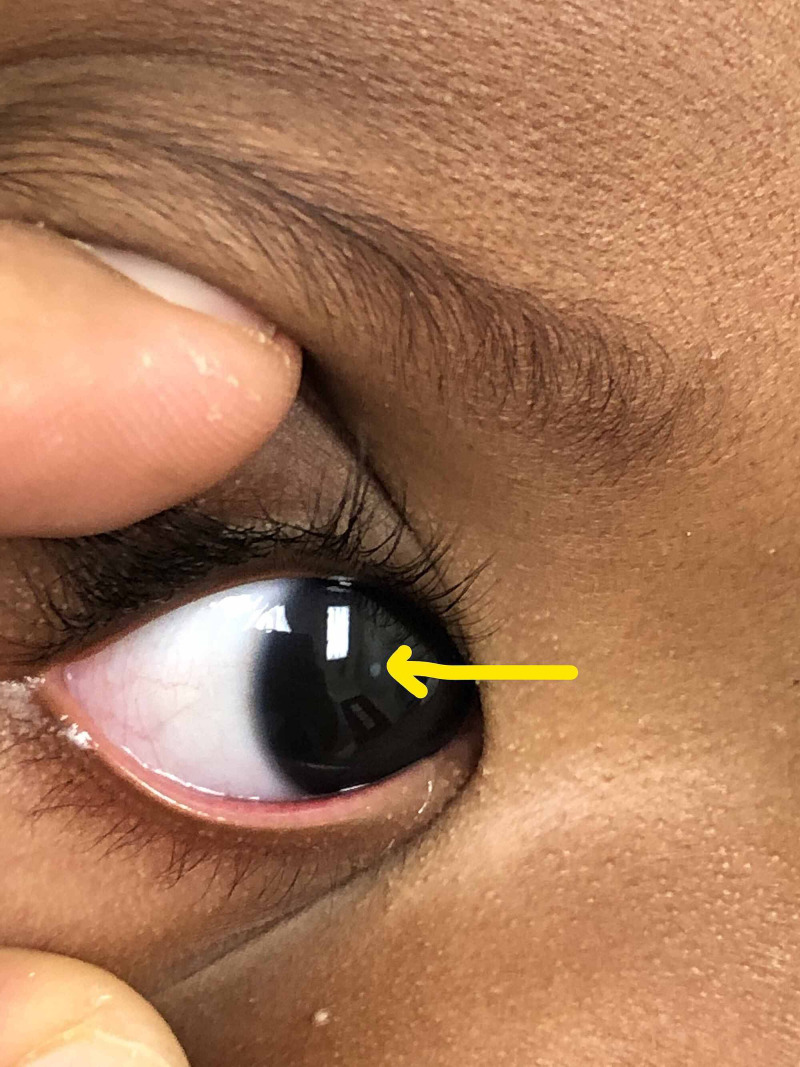
Leukocoria in the right eye

A nine-day-old male infant was brought to the pediatric clinic by his mother for evaluation of a white spot in his right eye. The mother had noted a whitish lesion in his right eye. She did not have any other concerns regarding the infant. He was born via an uncomplicated repeat cesarean section at full term. The mother’s prenatal serologies were negative. No known ocular diseases on review of family history (Figure 1).

On examination, the infant was well appearing and alert. He had no apparent dysmorphic features. Ophthalmic examination revealed that the pupils were round and equally reactive to light. The rest of the physical examination was within normal limits. No abnormalities of the lids, lashes, conjunctiva and sclera of either eye were noted. Extraocular movements were intact. The red reflex in the left eye was present and clear. The right pupil had a whitish appearing circular opacity.

The infant was referred to a pediatric ophthalmologist who evaluated him via fundoscopic examination and a diagnosis of a Mittendorf dot was confirmed. He was also found to have oblique myopic astigmatism of both eyes. Infant has been following up with the ophthalmologist without any current abnormalities of vision till nine months of age. He may require glasses as he grows older due to his myopic astigmatism.

## Discussion

Cataracts, glaucoma, retinal abnormalities, systemic diseases with ocular manifestations, retinoblastoma and high refractive errors can be picked up by checking for the red reflex. In children, cataract is one of the leading causes of treatable visual disability and blindness.

The hyaloid artery is present in the fetus and typically regresses completely by around 29 weeks of gestation [5]. The fetal remnant of the anterior attachment of the hyaloid artery is called the Mittendorf dot. It is a benign lesion. It appears as a small, circular opacity in the inferonasal quadrant of the posterior lens capsule. It does not cause visual disturbances [9]. Isolated Mittendorf dot does not require any treatment [10].

PHPV is typically unilateral and can present with a wide spectrum of clinical signs [11]. Microphthalmia, leukocoria and cataract are the most common clinical findings. There are three types of PHPV, i.e., anterior, posterior and a combination of the two [12]. Depending on the severity of the case, complications like glaucoma, retinal detachment, intraocular hemorrhage, uveitis and phthisis bulbi could occur [13]. Treatment may need to address complications like glaucoma and phthisis bulbi first. Lensectomy along with either an anterior or total vitrectomy and trabeculectomy as the usual surgical procedures are performed based on the clinical findings.

## Conclusions

A pediatrician’s evaluation of an infant for red reflexes is an easy screen that can be performed with the direct ophthalmoscopic examination. It helps identify several pathological conditions, the treatment of which in a timely fashion can help prevent blindness or loss of the eye. Though most of the causes of leukocoria require treatment, this case brings to light the Mittendorf dot, a benign condition that can cause this clinical finding that frequently leads to immediate concern.
